# The importance of science-informed policy and what the data really tell us about e-cigarettes

**DOI:** 10.1186/s13584-015-0021-z

**Published:** 2015-05-15

**Authors:** David B. Abrams, Raymond Niaura

**Affiliations:** Department of Health, Behavior and Society, Bloomberg School of Public Health, The Johns Hopkins University, 624 N. Broadway, Suite 280, Baltimore, MD 21205 USA; Department of Oncology, Lombardi Comprehensive Cancer Center, Georgetown University Medical Center, Washington, DC USA

## Abstract

A possible future end-game for cigarettes is explored in the context of the historical progress made to date by tobacco control. Despite good progress, there remains an urgent need to increase the use of proven tobacco control policies and practices for prevention and cessation. The problem is worse than previously thought and the 50^th^ anniversary United States Surgeon General’s report indicates the overwhelming majority of avoidable deaths are caused by combusting of tobacco, primarily cigarettes. The report highlights for the first time the addition of a harm minimization strategy to enhance proven tobacco control efforts and thus much more rapidly speed the obsolescence of cigarettes. Harm minimization can be two pronged. First, it can boost proven tobacco control polices to make cigarettes more expensive and less appealing and accessible to maximize the fact that cigarettes are orders of magnitude the most harmful of all tobacco delivery systems. Second, harm minimization can support use of substantially less harmful but appealing alternatives to substitute for lethal cigarettes for those users who are unable or unwilling to quit smoking. A future end-game might prudently manage emerging new products like e-cigarettes to help boost the difference in harm between them and lethal cigarettes. Harm minimization could help to accelerate the end of the century-long dominance of the cigarette in what has been called “the golden holocaust”. Rather than these emerging delivery devices being used to replace lethal cigarettes in what might be termed a David versus Goliath strategy to disrupt the status quo, there is also legitimate concern that these new products could undermine historically successful tobacco control efforts, especially youth prevention, if allowed free reign. What can the data really tell us about the potential for e-cigarettes to be helpful or harmful? The emerging but limited scientific evidence and the inherent methodological constraints in study designs, points to the need for caution in prematurely interpreting results in a manner that could mislead policymakers.

Rosen and Peled-Raz [1] provide an illuminating history of the lifesaving advances in tobacco control in Israel and examine future directions, including the rise of e-cigarettes. Israel like the United States (U.S.) is increasingly embracing more components of the Framework Convention for Tobacco Control [1, 2]. In figure 1, Rosen and Peled-Raz provide an impressive visual display of key policy and practice milestones, especially those implemented over the last 15 years [1]. In the U.S., the 2014 publication of the 50th anniversary edition of the Surgeon General’s Report includes similar policy and practice reviews, not all of which have been implemented at the highest possible levels either in the U.S. or in Israel: large tax increases; indoor air restrictions; education campaigns; no sales to minors; restricting marketing that targets youth; health insurance coverage for cessation treatment; protecting the unborn and pregnant moms from smoking; and protecting bystanders from environmental smoke pollution [3]. Making the next generation of youth tobacco-free and helping current smokers quit is a top priority of U.S tobacco control, yet progress will be slow if more is not done.

Despite best efforts in Israel to date, the sobering status in 2015 is the still unacceptable statistic of about 8,000 annual preventable deaths - and Israel is in good company, contributing its fair share to the about 1 billion deaths projected globally in the 21^st^ century. The deaths come overwhelmingly from one class of tobacco delivery product: the century-long behavior of inhaling the toxic smoke emanating from the combusting of cured tobacco leaves. Smoke delivered in the form of the mass produced, appealing, addictive, still *NOT* overly expensive, and continuously being engineered for optimal consumer satisfaction, but nonetheless lethal cigarette. Calls to eliminate cigarettes from the market are increasingly being made [2].

The astute historian Proctor calls the past “cigarette century” [4] the golden holocaust [5, 6]. In the U.S., cigarettes have prematurely killed over 20 million Americans, more lives lost than in all the American wars ever fought, from the War of Independence to the present. Proctor repeatedly calls for abolition. He defines a defective product as one that is not simply inherently risky, but risky by design: intentionally manufactured with an unacceptable level of risk to optimize the product’s chemosensory satisfaction and addiction propensity. Proctor’s plea falls on deaf ears.

As part of an end-game for cigarettes, Warner and others point out that strong economic disincentives can have a large impact on preventing youth initiation and reducing cigarette use, but the tax increases have to be large and regularly stepped up [7]. Policymakers would need the political courage to prioritize people’s lives over commercial profits in line with social justice. Overt explicit resistance, but more critically inaction and failure to challenge misguided resistance to prudent policy, means that vulnerable victims of lethal cigarette access, especially youth, are not protected from a defective consumer product. A silent majority and a few vocal supporters of a free market actually protect the economic interests of the Multinational Tobacco companies and put profits over saving people’s lives. More resources dedicated to public health and stronger enforcement of policies that minimize the harms of lethal cigarettes are sorely needed.

In the U.S. in 2009, in addition to state and federal tax increases (although still not nearly high enough), a new “user fee” was also implemented on cigarette sales. The fees fund the U.S. Food and Drug Administration (FDA) through a sustained infrastructure (the Center for Tobacco Products) for tobacco control regulatory oversight. The fees also fund research and ongoing national surveillance specific to timely and effective regulatory policymaking. Thus, another doubling of the tax on cigarettes and imposing a user fee could be powerful tools to prevent many more precious children alive today from starting to smoke and encourage smokers to quit.

Rosen and Peled-Raz remind Israel of the formidable challenges and ask for more resources to make future opportunities and dreams come true to protect all Israelis, both Jewish and Arab, both men and women. As in the U.S., there is a need for more financial resources to further strengthen science-informed policy and practice [1].

The U.S. Surgeon General’s 2014 report [3] also points out that more must be done in addition to the traditional proven tobacco control measures to hasten the end- game for tobacco [7]. For those who cannot or do not want to stop smoking, alternative less harmful products, in addition to approved medications and behavior therapy, should now be considered and prudently regulated. Specifically, and consistent with Warner’s [7] and with Rosen and Peled-Raz’ s [1] end-game examination of future directions, the U.S. Surgeon General suggested the addition of harm minimization strategies to be added to proven historical tobacco control strategies:

*“Death… is overwhelmingly caused by cigarettes and other combustibles… promotion of e-cigarettes and other innovative products is… likely to be beneficial where the appeal, accessibility and use of cigarettes are rapidly reduced*” (pp. 15–17) [3].

Harm minimization is a two-pronged strategy to heighten and not diminish the differences between the massively harmful cigarette and less harmful alternatives. The challenging question raised is: what is the best way to implement harm minimization in this rapidly changing landscape, as a pragmatic set of new tools to speed the obsolescence of lethal defective cigarettes? Is this a David versus Goliath strategy to disrupt the status quo of the powerful tobacco corporations? There are legitimate concerns about how to manage emerging products like e-cigarettes. The science is still too preliminary to be able to inform policy and practice with confidence. For example, we need to know how to ensure that any new reduced harm products are kept away from youth and used primarily to eliminate much more lethal cigarettes, rather than to perpetuate or promote continued use of cigarettes. Nonetheless, Rosen and Peled-Raz have set the stage for an exploration of future scenarios to save more lives [1]. Their exploration of end-games raises the thorny issue of what is known already and what is being interpreted from the science about the e-cigarette class of emerging products. The uncertainty and strong emotion in addressing this class of products must be considered in the context of the opportunity for policies to prudently manage the potential risks and benefits of these products without over or under reacting.

## Science, emotion and policy

Four centuries ago Sir Francis Bacon wrote: “human understanding is no dry light but receives infusions from the will and affections” [8]. When the stakes are high, as they are in the currently shifting tobacco control landscape, fears of unknown and hypothetical consequences can fuel strong emotion. Can such fears cloud a rational, scientific approach to health policy and mislead policymakers and the public? Is this now characteristic of the situation regarding the class of emerging products represented by e-cigarettes that may or may not contain nicotine (and more broadly other emerging alternative nicotine delivery systems - ANDS)? Bacon’s remarks remain pertinent when, 400 years later, scientists relinquish their dispassionate stance and make policy recommendations with strong conviction, despite strong disagreement within their own ranks about making premature conclusions derived from the same and oftentimes sparse data.

This commentary examines whether interpretation of evidence about ANDS is being clouded by emotion and past ideology, rather than a reliance on the scientific method and a rational, humble and cautious approach based on only preliminary evidence. ANDS represents a new class of products that is rapidly gaining in popularity and technical innovation. Knowledge regarding their use, safety and public health significance is accumulating, but has divided the tobacco control community. Some are legitimately nervous about ANDS' potentially deleterious impact on tobacco use behavior and others view ANDS as an alternative with potential to displace lethal cigarettes [9]. There are few facts available to policymakers to guide regulatory decisions at this early stage. But there are troubling trends in the interpretation of scant evidence and policy recommendations. Positions have been taken across the spectrum of the debate about whether ANDS can be helpful or harmful to public health. The discomfort is essentially one between an ideological framework that derives from the past 40 years of proven tobacco control strategies to eliminate all tobacco and nicotine use and those who wish to embrace the addition of a harm minimization approach to the traditional status quo framework.

Two brief examples based primarily on the current situation in America illustrate the nature of the ideological conflict; one is drawn from data on patterns of youth uptake and the other from data on adult cessation. In its general form, the debate can be framed as the challenge of constructing a new transformational science-based framework to exploit what could be one of the greatest opportunities of the 21^st^ century: To speed the end-game goal of eliminating tobacco-related deaths caused overwhelmingly by the inhalation of combustible tobacco products (primarily cigarettes and also cigars, hookah/waterpipe and related products). ANDS advocates believe that the products can literally speed the demise of combustible tobacco. Opponents claim these products are yet another wolf in sheep’s clothing, a Trojan horse that will help to attract and addict a new generation who will progress in droves to become lethal lifetime cigarette users. ANDS will also promote dual use and thus will maintain lethal cigarette use among current smokers and slow cessation.

It is premature to predict how the future will play out, but a key question is what is it that scientists, advocates, regulators and policymakers will actually do that helps or (unwittingly) hinders the speedy demise of the lethal cigarette [9]. Does ANDS represent a disruptive technological advance (a sheep in wolf’s clothing, rather than the wolf in sheep’s clothing so to speak - a gift horse and not a Trojan horse, perhaps) that will finally disrupt the deadly hold that cigarettes have had over a significant proportion of the world’s population for over a century and themselves spawned by the invention of the cigarette rolling machine [10, 11]? The first rolling machines invented in the 1880’s produced about 200 cigarettes per minute, the modern version produces 18,750 cigarettes per minute and can run 24 h a day, 7 days per week.

Our view is that ANDS represent a new set of tools with the potential to minimize harm related to combustible tobacco use [11] but only if they are prudently managed and marketed to adult smokers and only if the public is accurately and truthfully informed about them and how best to use them to eliminate combustible cigarette use. It must be stressed, however, that traditional tobacco control strategies and tactics to make lethal cigarettes less appealing, accessible and more expensive should continue in full – and perhaps, greater force to deter progression from ANDS to lethal combustibles. Nothing in this commentary should be interpreted as undermining current tobacco control efforts [2]. We propose that, because of the rapidly changing landscape, it is time to add a prudent and pragmatic harm minimization transformative framework to the traditional tobacco control views and interpretations of evidence used to guide practice and policy.

## Uptake and progression or cessation from lethal cigarettes?

### Uptake and progression

An increasing number of studies are being published based on similar observational designs from surveillance samples in the U.S., largely cross sectional but some longitudinal. The studies generally were not designed to look at the question of whether ANDS deters or promotes the use of lethal cigarettes. Many have included just one or two questions about ANDS use - typically lifetime (ever) use and past 30-day (experimental use but often called current use). ANDS critics claim the studies replicate one another and they conclude that their evidence shows that ANDS are dangerous, are likely a pathway into more lethal cigarette use and are undermining the progress made in tobacco control.

One of the most prominent of these studies illustrates common concerns about the conclusions drawn from many similar studies [12]. Using data from a large representative cross-sectional study of U.S. school students, Dutra and Glantz reported e-cigarette experimental use was associated with more use of lethal cigarettes [12]. Strong conclusions and policy recommendations were drawn, despite the correlational, rather than causal, nature of the study design which they did acknowledge but none the less stated: “Use of e-cigarettes does not discourage and may encourage, conventional cigarette use among U.S. adolescents” and “e-cigarette use is aggravating rather than ameliorating the tobacco epidemic among youths.” The data do not support the conclusions [13]. The survey data and design do not in any way document movement from e-cigarettes to combustible cigarettes. It’s equally plausible that use of combustible cigarettes leads to use of e-cigarettes, because they are perceived as a less harmful alternative for smokers who are not able or willing to go without smoking or are simply experimenting with a new and novel product out of curiosity. Finally, the Dutra and Glantz [12] noted a correlation of use of e-cigarettes with more time spent using tobacco products, which they believe “… call[s] into question claims that e-cigarettes are effective as smoking cessation aids.” But users of e-cigarettes also had higher intention to quit. This connection may indicate that e-cigarette users may have adopted e-cigarettes for harm reduction (i.e., to assist with quitting). In any case, the article cannot examine directionality.

Despite the commonly known and basic limitation of not inferring direction of causality from observational data collected at a single time point, the overwhelming majority of statements and policy recommendations (based not only on this study but also on several others of similar design) suggest that ANDS are a pathway into lethal cigarettes and undermine efforts to quit smoking. Together the emerging and similar studies appear to replicate one another and thus seem on the surface to strengthen what amounts to the same premature and possibly misleading conclusions. The findings of a correlation between use of ANDS and cigarettes may appear to be consistent, but none of the studies can prove a causal effect, much less confirm its direction; the correlation could also be indicative of underlying shared vulnerabilities that predispose experimenters to engage in a variety of risky behaviors, without one being a gateway to the others [13].

The context in which the study results were observed is also telling. According to data from the U.S. National Youth Tobacco Survey (NYTS), use of lethal cigarettes actually declined every year from 2011 to 2014 [14]. Therefore, if youth uptake of e-cigarettes were to represent a threat to public health, this downward trend would be slowed or reversed as a direct result of ANDS use. ANDS would have to be causally linked to either undermining cessation of lethal cigarettes and/or to enticing sufficiently large numbers of youth to progress to lethal cigarette use. To slow or reverse the downward trend in combustible use the net number of youth who otherwise would never have smoked cigarettes at all would have to increase, not simply just the youths who may now start with ANDS but who later would have been cigarette smokers anyway. In fact, while these trends ideally should be tracked up until about age 25 in a longitudinal cohort, the most recent cross-sectional NYTS 2014 data indicates e-cigarette experimental use increased from 2010 to 2014 from 1.5 % to 2.8 % to 4.5 % to 13.4 %. But over the same time period use of lethal cigarettes decreased steadily by about 10 % per year from 2011 to 2013 (15.8 % to 14.0 % to 12.7 %) and decreased even more rapidly to a record low of 9.2 % (a near 25 % reduction from 2013 to 2014) [14, 15].

It is too early to draw any conclusions and longitudinal data are needed over at least 5 or more years to test the reality of fears of uptake of ANDS directly leading to more uptake and progression to lethal cigarette daily than if they did not exist - and that excess progression would ultimately undermine public health. It is troubling that the strong negative conclusions and recommendations already made may already be clouding what the data really tell us. As Niaura et al. stated: “Cross-sectional surveys provide us with valuable descriptive information that prompt us to watch carefully how many youth are using tobacco products and e-cigarettes, but do not provide explanations for use” [13]. Prematurely over-interpreting or misinterpreting data, perhaps based on past ideology, excessive fear in a changing marketplace or human bias, does not help the cause of tobacco control or public health. Implying causal explanations in the absence of appropriate data can lead the health care community and policymakers down false paths on the road to relieving the horrific toll that lethal cigarettes impose on society.

### Cessation

Key issues regarding ANDS and public health impact also relate to their potential role in facilitating smoking cessation: whether they: (a) significantly minimize harm by substituting for lethal cigarettes; (b) have little effect on harm because smokers use both products without appreciable reduction in toxicant exposure; or (c) speed up or delay cessation in those who would otherwise have quit smoking altogether. In addition, even if ANDS are substantially lower in harm and can save lives, it must also be stressed that they are not harmless. Those ANDS products that contain nicotine and other additives and flavors do pose some risk and the risk is greater if they have poor quality control standards, are sold to minors or marketed in ways that appeal to youth and if the bottles of the liquid juice are sold without child resistant containers, accurate labels and clear warnings and instructions for safe handling. Thus the ideal for minimizing harms (to zero) remains the cardinal goal of tobacco control: no use of any tobacco or nicotine product at all.

Like the interpretations of the ANDS uptake data, some researchers and policymakers and an increasing number of similarly flawed research study designs and limited measures have also made strong statements that ANDS do not aid in cessation, are negatively associated with cessation and may undermine cessation efforts that could have been better accomplished by use of FDA-approved pharmacotherapies such as medical nicotine replacement – NRTs [16]. The evidence to date does not support this simple negative interpretation of their data. Several observational studies, for example, reported that any use of e-cigarettes is correlated with reduced cessation [17–19]. In some studies, smokers were not even asked whether they used e-cigarettes to help them quit or for how long they tried to use ANDS to quit, but simply were asked questions like: Did you ever use e-cigarettes in your lifetime? Without basic information about reasons for use and duration of use, these negative correlations are impossible to interpret with respect to helping or hindering lethal smoking cessation [20]. Other factors, especially selection bias, may also be at play [20]. For example, smokers who are, for whatever reasons, less able to quit, but are more motivated to try a new product, may be more likely to experiment with e-cigarettes, but either not be interested in cessation or may have had such prior difficulty with cessation that they are disproportionately likely to fail again [20].

Two randomized controlled trials with early and technologically inefficient e-cigarette models still showed that ANDS are modestly effective in helping some smokers to quit or reduce lethal cigarette consumption [21, 22]. Bullen *et al.* [21] compared ANDS to nicotine replacement therapy (NRT) and, using the six month point prevalence outcome criterion employed in the meta-analysis for the U.S. Clinical Guidelines [23], reported an encouraging 21.1 % cessation rate for ANDS and 15.6 % for NRT (not a statistically significant difference), indicating ANDS are at least as effective as NRTs. A U.S. longitudinal observational study found after 2–3 years, intensive e-cigarette users (used daily for at least a month for purposes of quitting) had the highest rate of smoking cessation (20.4 %), compared to intermittent users (more than once or twice but not daily for a month or more; 8.5 %) and non-users (12.4 %) [24]. Smokers who used ANDS for at least a month were six times more likely to be abstinent 2 years later [24]. Another large cross-sectional survey in the United Kingdom (U.K.) found ANDS users were more likely to report abstinence than either those who used NRT or no aid [24]. In the U.K., ANDS use for cessation has also surpassed use of NRT’s making ANDS potential for larger scale population impact quite promising. ANDS may be more appealing, convenient and less expensive to millions more smokers than conventional smoking cessation aids.

More research – especially high quality randomized controlled trials is needed to further determine whether and how ANDS can be an effective cigarette cessation or harm minimization tool [25, 26]. ANDS have also been shown to reliably decrease adverse symptoms related to tobacco abstinence (*e.g.*, cravings and urges to smoke, irritability) [27–31]. The U.K approach to lightly regulate ANDS and encourage their use for lethal cigarette cessation is an interesting policy to be watched in the coming years. However without convincing data some countries (*e.g.*, Brazil) have outright banned ANDS products while others have permitted products to be sold without nicotine (*e.g.*, Canada, Australia). The different experiences will accelerate global learning about very different policies based on sparse data.

## Research challenges

Once again, what the data really tell us about ANDS and cessation of lethal cigarettes stands in sharp contrast to the strongly stated convictions from a few scientists: that ANDS impede smoking cessation and can harm tobacco control efforts. As Bacon’s 400-year old observation reminds us, science calls for more rigor and less emotion. Keeping an open mind to new innovation is important despite legitimate anxieties. The scientific method calls for acknowledging equally and skeptically the actual data for and against ANDS supporting uptake and progression to lethal cigarettes and in evaluating ANDS’ efficacy for lethal cigarette cessation [20, 26, 32, 33]. Net public health impact of ANDS is a complex interaction of many factors at multiple levels of influence [9]. Systems thinking and simulation modeling tools [34] will be needed along with more informative data before we will be able to say how best to maximize the benefits of ANDS as a disruptive technology [35] and minimize the hypothetical harms of ANDS to the population as a whole, users and non users, especially youth [2, 10, 11, 26, 36].

## Conclusion

If properly regulated and responsibly made and marketed, especially by independent manufacturers who have less conflict as Big tobacco does by also selling lethal cigarettes, ANDS have the potential to improve the public health. Together with traditional tobacco control strategies ANDS can speed the demise of the lethal cigarette by deterring progression to lethal cigarettes among non-users and by providing smokers who do not want to, or have tried and cannot “just quit”, with a substantially safer alternative - without causing the suffering from the devastating health consequences imposed on them by the need to inhale toxic cigarette smoke. As Dr. Russell stated so prophetically 40 years ago and which is still true today, “people smoke for the nicotine but die from the tars” [37]. ANDS raise legitimate fears of possible risks and these need to be watched and managed, but misinterpreting or selectively presenting negative preliminary data does not serve the public health. The precautionary principle is potentially violated if ANDS are prematurely demonized and are then seen by the public as simply another dangerous tobacco product instead of a way to make lethal cigarettes obsolete. Then we may well have missed the tobacco control opportunity of the century [35].

### Commentary on

Rosen LJ, Peled-Raz M. Tobacco Policy in Israel: 1948–2014 and beyond. Journal of Health Policy Research 2015.

### Endnote

Parts of this paper include material adapted from the author’s contributions to the 2014 *The Health Consequences of Smoking—50 Years of Progress: A Report of the Surgeon General* and other recent commentaries and publications on e-cigarettes.
